# A novel RNA sequencing‐based miRNA signature predicts with recurrence and outcome of hepatocellular carcinoma

**DOI:** 10.1002/1878-0261.12315

**Published:** 2018-05-21

**Authors:** Fumao Bai, Huaibin Zhou, Mengni Ma, Chen Guan, Jianxin Lyu, Qing H. Meng

**Affiliations:** ^1^ Key Laboratory of Laboratory Medicine Ministry of Education of China Zhejiang Provincial Key Laboratory of Medical Genetics School of Laboratory Medicine and Life Sciences Wenzhou Medical University China; ^2^ Department of Laboratory Medicine The University of Texas MD Anderson Cancer Center Houston TX USA

**Keywords:** biomarkers, disease recurrence, hepatocellular carcinoma, microRNA, risk score

## Abstract

Hepatocellular carcinoma (HCC) is the fifth most common type of cancer and the second leading cause of cancer‐related deaths worldwide. Given that the rate of HCC recurrence 5 years after liver resection is as high as 70%, patient with HCC typically has a poor outcome. A biomarker or set of biomarkers that could predict disease recurrence would have a substantial clinical impact, allowing earlier detection of recurrence and more effective treatment. With the aim of identifying a new microRNA (miRNA) signature associated with HCC recurrence, we analyzed data on 306 patients with HCC for whom both miRNA expression profiles and complete clinical information were available from The Cancer Genome Atlas database. Through this analysis, we identified a six‐miRNA signature that could effectively predict patients’ recurrence risk; the high‐risk and low‐risk groups had significantly different recurrence‐free survival rates. Time‐dependent receiver operating characteristic analysis indicated that this signature had a good predictive performance. Multivariable Cox regression and stratified analyses demonstrated that the six‐miRNA signature was independent of other clinical features. Functional enrichment analysis of the gene targets of the six prognostic miRNA indicated enrichment mainly in cancer‐related pathways and important cell biological processes. Our results support use of this six‐miRNA signature as an independent factor for predicting recurrence and outcome of patients with HCC.

**Abbreviations**
AUC: area under the curve
CI: confidence interval
CRC: colorectal cancer
GO: gene ontology
HCC: hepatocellular carcinoma
HR: hazard ratio
KEGG: Kyoto Encyclopedia of Genes and Genomes Pathway
OS: overall survival
RFS: recurrence‐free survival
ROC: receiver operating characteristic
TCGA: The Cancer Genome Atlas

## 1 Introduction

Hepatocellular carcinoma (HCC), which accounts for 90% of liver cancers, is the fifth most common type of cancer and the second leading cause of cancer‐related deaths worldwide. In 2012, an estimated 782 500 new cases of HCC were diagnosed and 745 500 deaths were attributable to HCC worldwide (Ao *et al*., 2017; El‐Serag, 2011; Torre *et al*., 2015). The therapeutic approaches to HCC include liver resection, transplantation, ablation, chemoembolization, and targeted therapy with sorafenib (Mokdad *et al*., 2016). Liver resection is considered the first choice in HCC treatment, but the recurrence rate at 5 years after liver resection exceeds 70% (Vilarinho and Calvisi, 2014). Although postoperative chemotherapy is reported to be effective in some patients with recurrence, its efficacy remains controversial (Hashimoto *et al*., 2005).

Because of this high rate of recurrence (Vilarinho and Calvisi, 2014) and the challenge of treating recurrent HCC (Poon, 2011), the prognosis of HCC is still very poor. One or more molecular biomarkers that accurately predict recurrence would be of great clinical significance. Several studies have tried to identify such biomarkers (Jin *et al*., 2013; Kim *et al*., 2014; Yang *et al*., 2017), but an early and reliable predictor that can be applied readily to clinical practice is still lacking, and identifying patients at high risk for recurrence is currently beyond our reach. The ability to distinguish patients with HCC at high risk of recurrence from those at low risk may lead to development of more effective therapeutic regimens and personalized therapies, which hold the promise to prolong survival and improve overall outcome.

miRNA are small noncoding RNA 18–25 nucleotides in length. Like long noncoding RNA and protein‐coding genes, miRNA can function as tumor suppressors or oncogenes during tumor progression (Bartel, 2004; Kwak *et al*., 2010). The mechanism through which miRNA contribute to cancer development is direct binding to the 3′‐untranslated region of mRNA to inhibit their translation (Bartel, 2009). There are some reports that miRNA can bind to the 5′‐untranslated region of mRNA (Chaluvally‐Raghavan *et al*., 2016), suggesting their complicated biological function. Increasing evidence suggests that miRNA play important roles in various biological processes, such as cellular development, metabolism, and proliferation (Ambros, 2004; Guo *et al*., 2015). In particular, it is believed that miRNA drive the pathogenesis and progression of cancer. Our previous studies and findings from other investigators have demonstrated associations of miRNA with a variety of cancers (Bae *et al*., 2015; Li *et al*., 2016, 2017; Szabo and Bala, 2013; Zhang *et al*., 2016). This justifies a new approach of investigating miRNA as potential cancer biomarkers. The prognostic value of various miRNA signatures has been reported in different types of cancers, such as colon cancer (Zhang *et al*., 2013), malignant pleural mesothelioma (Kirschner *et al*., 2015), lung cancer (Yu *et al*., 2008), and ovarian cancer (Bagnoli *et al*., 2016). Recently, miRNA associated with prognosis were reported in HCC. For instance, miR‐139, miR‐26, and miR‐140 have been reported to be associated with overall survival (OS) of patients with HCC (Ji *et al*., 2009; Wong *et al*., 2011; Yang *et al*., 2013). However, the prognostic value of miRNA in predicting the risk of disease recurrence in patients with HCC has not been fully elucidated.

Our purpose in this study was to identify miRNA that might serve as clinical biomarkers of HCC recurrence. Using miRNA sequencing data on HCC patients from The Cancer Genome Atlas (TCGA), we constructed a novel six‐miRNA signature that effectively identified patients at high risk of recurrence. Such a signature might be helpful in guiding development of novel, more effective individualized therapies, and improving clinical outcome of patients with HCC.

## 2 Materials and methods

### 2.1 TCGA miRNA dataset and patient information

MiRNA expression profiles and clinical data on 377 patients with liver cancer (seven hepatocholangiocarcinoma [mixed], three fibrolamellar carcinoma, and 367 HCC) were downloaded from the TCGA data portal (https://tcga-data.nci.nih.gov/). Patients chosen for model building met the following criteria: (a) histologic diagnosis of HCC; (b) both miRNA expression profile and complete clinicopathological and follow‐up data available; and (c) recurrence‐free survival (RFS) of more than 30 days and less than 3000 days. After filtering, a total of 306 patients were enrolled for further analysis. In addition, miRNA expression data for 50 adjacent noncancer tissues were also retrieved, and the expression of the prognostic miRNA between nontumor tissues and tumor tissues was compared. Those miRNA with an average count > 1 across all patients were kept in the expression profiles and normalized using the R/Bioconductor package of edgeR (Robinson *et al*., 2010). Because the data were all publicly available through the TCGA project, approval by our institutional ethics committees was not required. This study meets the publication guidelines provided by TCGA.

### 2.2 Prognostic model construction and statistical analysis

In the training set, a univariable Cox analysis was first performed to screen for miRNA correlated with RFS (*P* < 0.01). A multivariable Cox analysis was performed, from which six miRNA (miR‐210, miR‐550a‐1, miR‐3199‐2, miR‐4732, miR‐22, and miR‐139) were selected as independently associated with the RFS of patients with HCC (*P* < 0.01). We then devised a risk score that was a linear combination of the expression of these six miRNA and the multivariable Cox regression coefficients. The risk score for each patient was calculated as follows: Risk score = 0.215 × log2(Exp_miR‐210_) + 0.376 × log2(Exp_miR‐550a‐1_) − 0.113 × log2(Exp_miR‐3199‐2_) − 0.069 × log2(Exp_miR‐4732_) − 0.469 × log2(Exp_miR‐22_) − 0.311 × log2(Exp_miR‐139_). The risk scores of patients in each set were ranked ascendingly. The median risk score derived from the training set was used as a cutoff to separate the patients in each set into high‐ and low‐risk groups. Differences in RFS and OS between the high‐ and low‐risk groups were demonstrated by Kaplan–Meier curves and the log‐rank test. The chi‐square test was used to compare the difference in recurrence status between high‐ and low‐risk groups. The prognostic performance was measured using the area under the curve (AUC) derived from time‐dependent receiver operating characteristic (ROC) analysis, and the accuracy of the risk score to predict RFS at 1, 3, and 5 years and OS at 3, 5, and 7 years was assessed. To evaluate the independence of the miRNA signature as a predictor, multivariable Cox proportional hazards regression analysis was performed, where the RFS or OS was set as the dependent variable and miRNA signature as covariable together with other clinical features, including patient sex and age at diagnosis, tumor stage and grade, and TNM stage. All statistical analyses were conducted using R language (Version 3.3.3). Survival curves and ROC curves were generated by the ‘survminer’ (Kassambara and Kosinski, 2017), ‘survival’ (Therneau, 1994), and ‘survivalROC’ (Heagerty *et al*., 2000) packages.

### 2.3 Bioinformatic analysis of miRNA target genes and pathways

Potential gene targets of the six prognostic miRNA were obtained from miRTarBase (Hsu *et al*., 2014), an experimentally validated miRNA‐target interactions database. For the analysis, 1113 target mRNA with experimental evidence indicating the targeting regulatory relations were chosen. The DAVID Bioinformatics Tool (Huang da *et al*., 2009) (https://david.ncifcrf.gov/, version 6.8) was used to analyze these mRNA for gene ontology (GO, biological processes) and KEGG pathway enrichment. In this study, GO terms and KEGG pathways with *P* < 0.05 were considered significantly enriched function annotations.

## 3 Results

### 3.1 HCC patients in training and testing sets

For this study, we analyzed data on a total of 306 patients from the TCGA HCC database for model construction. These patients were randomly and evenly divided into a training set (*n* = 153) and a testing set (*n* = 153). Baseline demographic and clinical characteristics of the two groups did not differ significantly (all *P* > 0.05). These characteristics are summarized in Table S1.

### 3.2 MiRNA associated with recurrence‐free survival of HCC patients in training set

**Figure 1:**
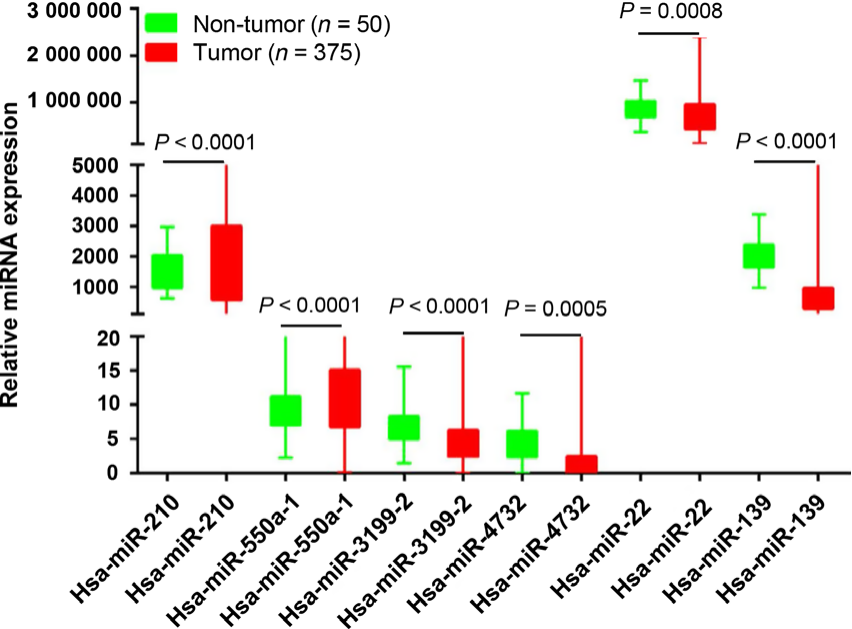
Expression of the six prognostic miRNA in HCC tumor and nontumor tissues. Expression of the six miRNA identified by multivariable Cox regression analysis was determined in tumor and corresponding nontumor tissues. Differences between tumor and nontumor tissues were analyzed by two‐sided Student's *t*‐test to determine significance. *P* < 0.05 was considered statistically significant. The boxplot shows the range (lower and upper whisker), the first quartile (lower hinge), and the third quartile (upper hinge). The result was graphed by graphpad prism 6.0 (GraphPad Software, Inc., San Diego, CA, USA).

To define the association of miRNA with HCC recurrence, we used univariable Cox regression analysis to identify 13 miRNA that were significantly associated with RFS of patients with HCC (*P* < 0.01). From these 13 miRNA, a multivariable Cox regression analysis identified six as exhibiting significant prognostic value (*P* < 0.01) (Table 1). Of the six, four (miR‐3199‐2, miR‐4732, miR‐22, and miR‐139) showed negative regression coefficients, implying that these miRNA play protective roles against recurrence and thus were considered to signal a low risk of recurrence. The remaining two miRNA (miR‐210 and miR‐550a‐1) showed positive regression coefficients, indicating that they are associated with high risk of recurrence. Expression of miR‐210 and miR‐550a‐1 was upregulated in HCC tissues compared to nontumor tissues, while miR‐3199‐2, miR‐4732, miR‐22, and miR‐139 were downregulated (Fig. 1).

**Table 1:** MiRNA associated with recurrence of HCC in the training set

Gene symbol	Coeff^a^	Type^b^	HR	low95	high95	*P*‐value^c^
has‐miR‐210	0.2150	Risky	1.2404	1.0658	1.4435	0.0054
has‐miR‐550a‐1	0.3760	Risky	1.4564	1.0982	1.9313	0.0090
has‐miR‐3199‐2	−0.1130	Protective	0.8935	0.8213	0.9721	0.0089
has‐miR‐4732	−0.0690	Protective	0.9330	0.8863	0.9823	0.0082
has‐miR‐22	−0.4690	Protective	0.6256	0.4791	0.8170	0.0006
has‐miR‐139	−0.3110	Protective	0.7324	0.6035	0.8887	0.0016

^a^Coefficients derived from multivariable Cox regression analysis. ^b^Types included protective (low risk) and risky (high risk). ^c^ *P*‐values obtained from multivariable Cox regression analysis.

### 3.3 Construction of miRNA signature and calculation of risk score in training set

**Figure 2:**
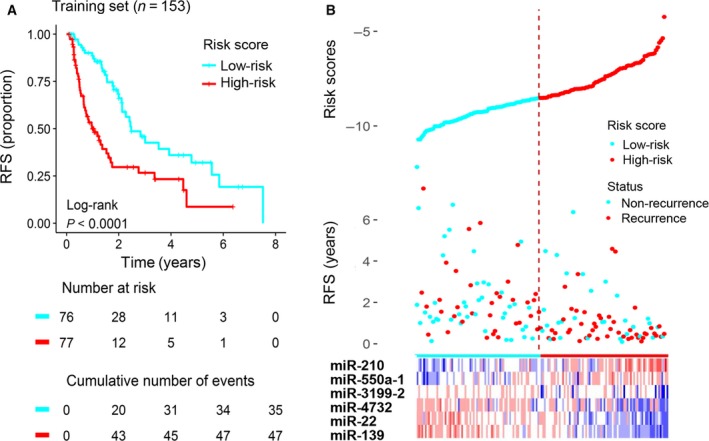
Construction of the six‐miRNA signature in the training set for determining HCC recurrence risk. (A) The training set was subjected to Kaplan–Meier analysis to compare RFS between patients in the high‐risk group and those in the low‐risk group. (B) The distribution of risk score, RFS, and recurrence status and the prognostic miRNA expression patterns for the 153 patients in the training set. The risk scores are arranged in ascending order from left to right. The number below the curve represents the number of the patients in the high‐ and low‐risk group. The ‘+’ symbols in the panel indicate censored data.

To facilitate the application of these miRNA in clinical practice, we designed a risk prediction formula based on the six identified prognostic miRNA (see Section 2) and calculated the risk score for each patient in the training set. The 153 patients with HCC in the training set were assigned to the high‐risk group (*n* = 77) or the low‐risk group (*n* = 76) by their risk scores; the cutoff between the groups was the median risk score (−8.65). Kaplan–Meier analysis showed that patients in the high‐risk group had a worse outcome than those in the low‐risk group (median survival 1.05 years vs. 2.47 years, log‐rank test, *P* < 0.0001; Fig. 2A).

The distributions of the risk scores, RFS, recurrence status, and corresponding miRNA expression profiles of the 153 patients in the training set are shown in Fig. 2B. The protective miRNA (miR‐3199‐2, miR‐4732, miR‐22, and miR‐139) tended to be more highly expressed in the low‐risk group, while the remaining miRNA (miR‐210 and miR‐550a‐1) were more highly expressed in the high‐risk group. Moreover, the high‐risk group had more recurrences than the low‐risk group: the high‐risk group comprised 47 patients with recurrence and 30 patients without recurrence, whereas the low‐risk group comprised 35 patients with recurrence and 41 patients without recurrence. However, this difference in recurrence frequency is marginally significant (chi‐square test, *P* = 0.06).

### 3.4 Validation of six‐miRNA signature in the testing set and entire TCGA set

**Figure 3:**
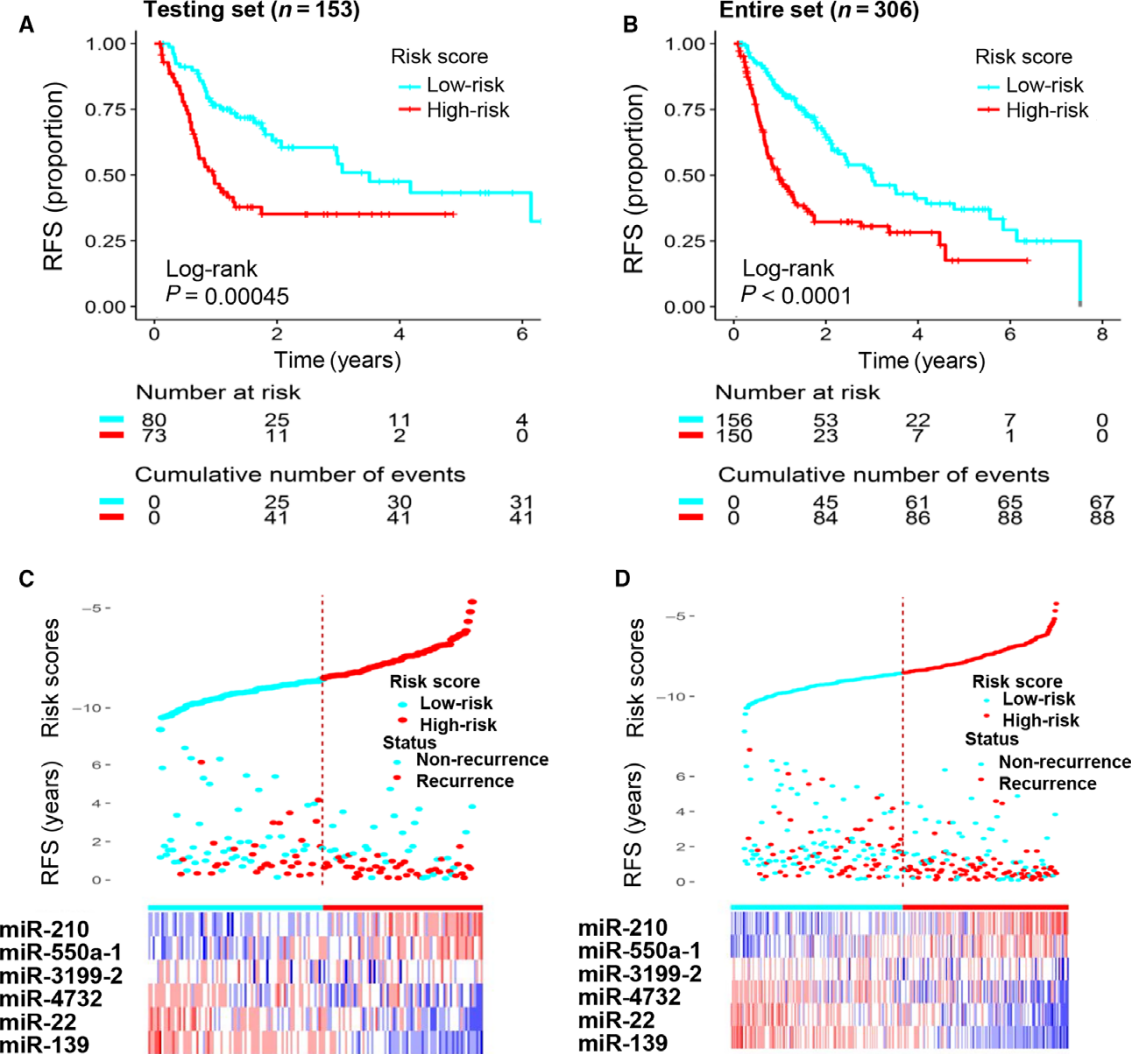
Validation of the prognostic value of the six‐miRNA signature in the testing and entire TCGA sets. (A) The testing set was subjected to Kaplan–Meier analysis to compare RFS between patients in the high‐risk group and patients in the low‐risk group. (B) The entire set was subjected to Kaplan–Meier analysis to compare RFS between patients in the high‐risk group and patients in the low‐risk group. (C) The distribution of risk scores, RFS, and recurrence status and the prognostic miRNA expression patterns for the 153 patients in the testing set. (D) The distribution of risk scores, RFS, and recurrence status and the prognostic miRNA expression patterns for the 306 patients in the entire set. The risk scores are arranged in ascending order from left to right. The number below the curve represents the number of the patients in the high‐ and low‐risk group. The ‘+’ symbols in the panel indicate censored data.

Using the same risk score formula and threshold value used in the training set, patients in the testing set (*n* = 153) and entire TCGA set (*n* = 306) were classified into high‐risk groups and low‐risk groups. Kaplan–Meier analysis showed that, in the testing set, patients in the high‐risk group (*n* = 73) had far shorter RFS than those in the low‐risk group (*n* = 80; median 0.95 years vs. 3.50 years; log‐rank test, *P* = 0.00045; Fig. 3A). Similar results were observed for the entire TCGA set (median 0.97 years vs. 2.99 years; log‐rank test, *P* < 0.0001; Fig. 3B).

The distributions of the risk scores, RFS, recurrence status, and corresponding miRNA signature expression profiles of patients in the testing set and the entire set are shown in Fig. 3C,D (ranked according to increasing risk scores). The results are consistent with the results obtained from the training set: The protective miRNA were upregulated in the low‐risk group and downregulated in the high‐risk group, while the risky miRNA were downregulated in the low‐risk group and upregulated in the high‐risk group. Moreover, most of the patients with disease recurrence in both sets were clustered in the high‐risk group. In the testing set, the high‐risk group comprised 41 patients with recurrence and 32 patients without recurrence, whereas the low‐risk group comprised 32 with recurrence and 48 without recurrence. The difference in recurrence status is statistically significance (chi‐square test, *P* = 0.046). For the entire TCGA set, the high‐risk group comprised 88 patients with recurrence and 62 patients without recurrence, while the low‐risk group comprised 67 patients with recurrence and 89 patients without recurrence. The difference in recurrence status between the two groups is significant (chi‐square test, *P* = 0.006).

### 3.5 Evaluation of the predictive performance of the six‐miRNA signature

**Figure 4:**
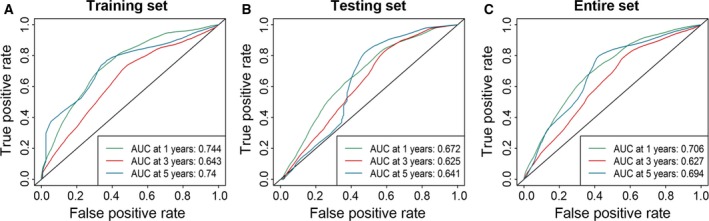
Performance assessment of the six‐miRNA signature by survival ROC analysis. ROC analysis of the six‐miRNA signature for prediction of recurrence risk at 1, 3, and 5 years in the training set (A), the testing set (B), and the entire set (C).

To evaluate the prognostic performance of the six‐miRNA signature, we performed a time‐dependent ROC curve analysis. The results showed that the six‐miRNA signature achieved AUC values of 0.744, 0.643, and 0.740 for predicting recurrence in the training set at 1, 3, and 5 years (Fig. 4A). The AUC values were 0.672, 0.625, and 0.641 for the testing set (Fig. 4B) and 0.706, 0.627, and 0.694 for the entire set (Fig. 4C). All of the AUC values exceed 0.6. These results suggested that our six‐miRNA signature performed well for prediction of disease course in patients with HCC.

### 3.6 Multivariable Cox regression analysis and stratified survival analysis

We assessed whether the miRNA signature maintains its prognostic value within the context of other clinical features. The results of multivariable Cox regression analysis show that the miRNA signature maintained independence in predicting the RFS of patients with HCC in the training set (hazard ratio [HR] = 1.62, 95% confidence interval [CI] = 1.346–1.966, *P* < 0.0001), the testing set (HR = 1.38, 95%CI = 1.101–1.701, *P* = 0.004), and the entire set (HR = 1.48, 95%CI = 1.298–1.696, *P* < 0.0001; Table 2).

**Table 2:** Multivariable Cox regression analysis of RFS in HCC patients in the training, testing, and entire sets

Characteristic	Multivariable analysis		
	HR	95% CI of HR	*P*‐value
Training set (*n* = 153)			
Risk score	1.623	1.346–1.966	**< 0.0001**
Sex, female/male	1.008	0.625–1.625	0.974
Age (years), ≥ 65/< 65	0.767	0.4634–1.271	0.304
Tumor stage, III.IV/I.II	15.280	1.579–147.89	**0.019**
Tumor grade, III.IV/I.II	0.897	0.552–1.457	0.660
T stage, T3.T4/T1.T2	0.124	0.013–1.178	0.069
N stage, non‐N0/N0	1.665	0.906–3.059	0.101
M stage, non‐M0/M0	0.952	0.505–1.792	0.878
Testing set (*n* = 153)			
Risk score	1.378	1.101–1.701	**0.004**
Sex, female/male	0.580	0.333–1.011	0.055
Age (years), ≥ 65/< 65	1.211	0.721–2.033	0.470
Tumor stage, III.IV/I.II	1.330	0.158–11.21	0.793
Tumor grade, III.IV/I.II	1.362	0.826–2.240	0.227
T stage, T3.T4/T1.T2	2.468	0.211–16.073	0.581
N stage, non‐N0/N0	0.843	0.376–1.582	0.479
M stage, non‐M0/M0	1.022	0.631–2.298	0.574
Entire set (n = 306)			
Risk score	1.484	1.298–1.696	**< 0.0001**
Sex, female/male	0.739	0.514–1.061	0.101
Age, years, ≥ 65/< 65	0.916	0.647–1.296	0.621
Tumor stage, III.IV/I.II	1.625	0.374–7.061	0.517
Tumor grade, III.IV/I.II	1.074	0.766–1.504	0.680
T stage, T3.T4/T1.T2	1.230	0.278–5.441	0.785
N stage, non‐N0/N0	1.028	0.661–1.598	0.904
M stage, non‐M0/M0	1.239	0.803–1.910	0.332

Tumor grade: neoplasm histologic grade; Tumor stage: AJCC pathological stage; T stage: tumor size; N stage: lymph node involvement; M stage: metastasis status. Values in bold indicate they are statistically different.

**Figure 5:**
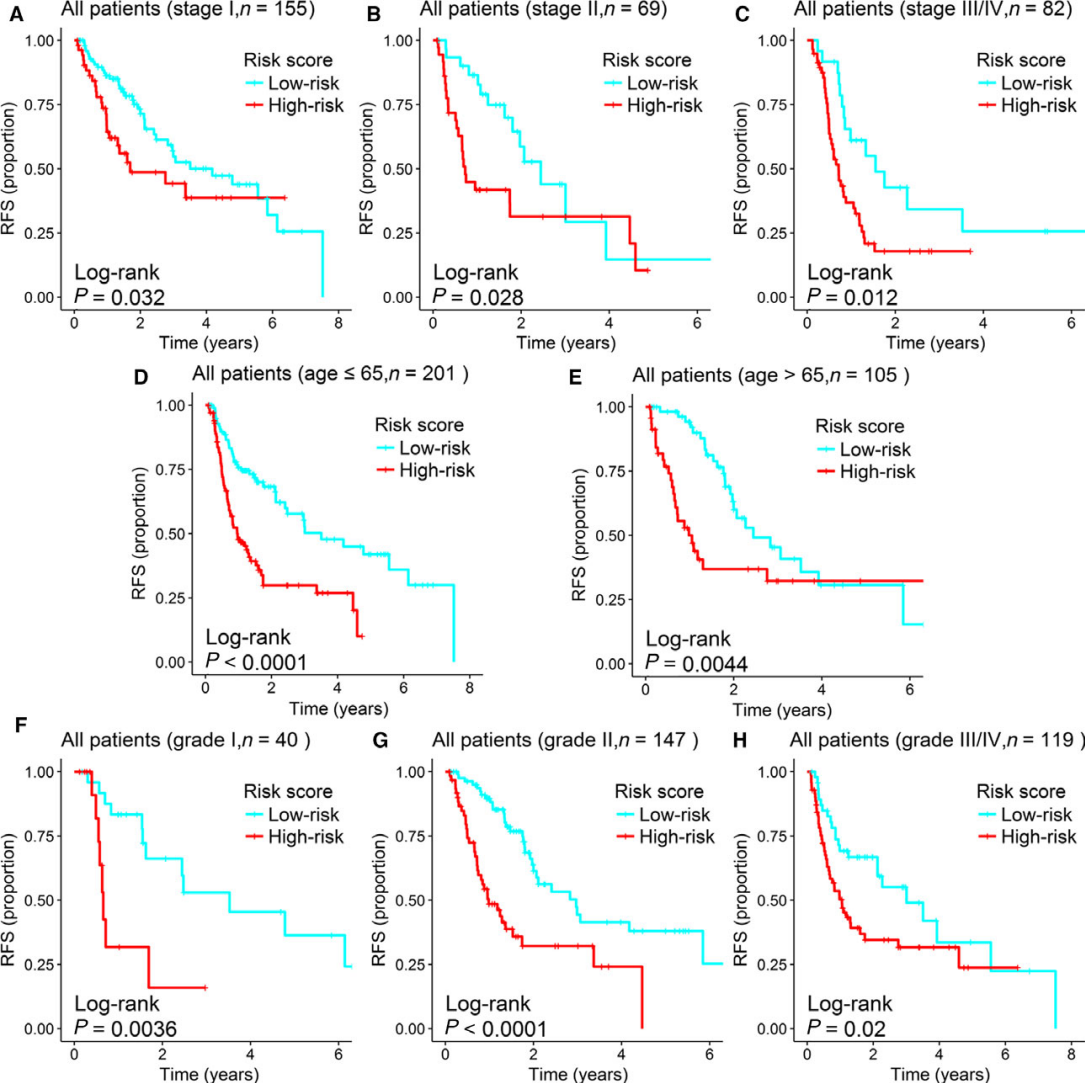
Survival analysis of all HCC patients stratified by stage, age, and tumor grade. Survival analysis compared RFS by recurrence risk (high vs. low) stratified by clinical characteristics. (A) Kaplan–Meier curves for patients with stage I HCC (n = 155). (B) Kaplan–Meier curves for the patients with stage II HCC (*n* = 69). (C) Kaplan–Meier curves for patients with stage III/IV HCC (*n* = 82). (D) Kaplan–Meier curves for patients aged 65 years or younger (*n *= 201). (E) Kaplan–Meier curves for patients aged older than 65 years (*n* = 105). (F) Kaplan–Meier curves for patients with grade I tumor (*n* = 40). (G) Kaplan–Meier curves for patients with grade II tumor (*n* = 147). (H) Kaplan–Meier curves for patients with grade III/IV tumor (*n* = 119). The ‘+’ symbols in the panel indicate censored data.

Because tumor stage was significant in the multivariable analysis in the training set, we conducted a stratified analysis with this clinical parameter. All 306 patients were stratified by tumor stage into a stage I dataset (*n* = 155), a stage II dataset (*n* = 69), and a stage III/IV dataset (*n* = 82). Using the six‐miRNA signature, patients in the stage I dataset were classified into a high‐risk group (*n* = 54) or a low‐risk group (*n* = 101); these groups had significantly different RFS (median 1.69 years vs. 4.17 years; log‐rank test, *P* = 0.0320; Fig. 5A). Likewise, the patients in the stage II dataset also were classified into a high‐risk group (*n* = 38) and a low‐risk group (*n* = 31), which also differed significantly in RFS (median 0.73 years vs. 2.44 years; log‐rank test, *P* = 0.028; Fig. 5B). Similarly, patients in the stage III/IV dataset could be subdivided into a high‐risk group (*n* = 58) with shorter RFS and a low‐risk group (*n* = 24) with significantly longer RFS (median 0.71 years vs. 1.55 years; log‐rank test, *P* = 0.012; Fig. 5C).

We also performed stratification analysis with the other two clinical features: age and tumor grade. All 306 patients were stratified by age into a younger dataset (age ≤ 65 years, *n* = 201) and an older dataset (age > 65 years, *n* = 105). Patients in the younger dataset could be stratified into a high‐risk group (*n* = 102) and a low‐risk group (*n* = 99) with significantly different RFS (median 0.97 years vs. 3.50 years; log‐rank test, *P *< 0.0001; Fig. 5D); a similar result was obtained in the older dataset (median 0.98 years vs. 2.44 years; log‐rank test, *P* = 0.0044; Fig. 5E). The prognostic performance of the six‐miRNA signature was similar when the entire set was stratified by tumor grade. The RFS of patients in the high‐risk group was significantly shorter than that of the low‐risk group in all three datasets (grade I dataset, median 0.66 years vs. 3.52 years, log‐rank test, *P* = 0.0036, Fig. 5F; grade II dataset, median 0.98 years vs. 2.97 years, log‐rank test, *P* < 0.0001, Fig. 5G; grade III/IV dataset, median 1.05 years vs. 3.01 years, log‐rank test, *P* = 0.02, Fig. 5H). When the same analysis with these three clinical variables was applied to the training set and the testing set, Kaplan–Meier curves of the high‐ and low‐risk groups were notably different, although the difference did not reach statistical significance in some subgroups. In those cases, a trend toward worse outcomes for patients with a high‐risk score was observed. The results of stratified analysis in the training and testing sets are shown in Figs S1 and S2.

### 3.7 Relationship between the miRNA signature and overall survival

Besides the association with RFS, we also analyzed the relationship between the miRNA signature and the OS of patients with HCC. Consistent with the RFS results, the results of this Kaplan–Meier analysis revealed that patients with a higher risk score had shorter OS than those with a lower risk score. The difference in OS between the two groups was statistically significant for the training set (*P* = 0.0062), the testing set (*P* < 0.0001), and the entire set (*P* < 0.0001; Fig. S3). Results of the multivariable Cox regression analysis (Table S2) and the data stratification analysis (Figs S4–S6) showed that the miRNA signature was independent of other clinical features in predicting OS. The AUC values of time‐dependent ROC curves were 0.658, 0.635, and 0.612 at 3, 5, and 7 years, respectively, for the training set. The AUC values were 0.752, 0.763, and 0.804 for the testing set and 0.709, 0.694, and 0.723 for the entire set; overall, the values ranged from 0.612 to 0.804 (Fig. S7).

### 3.8 Functional enrichment analysis of predicted target genes of prognostic miRNA

**Figure 6:**
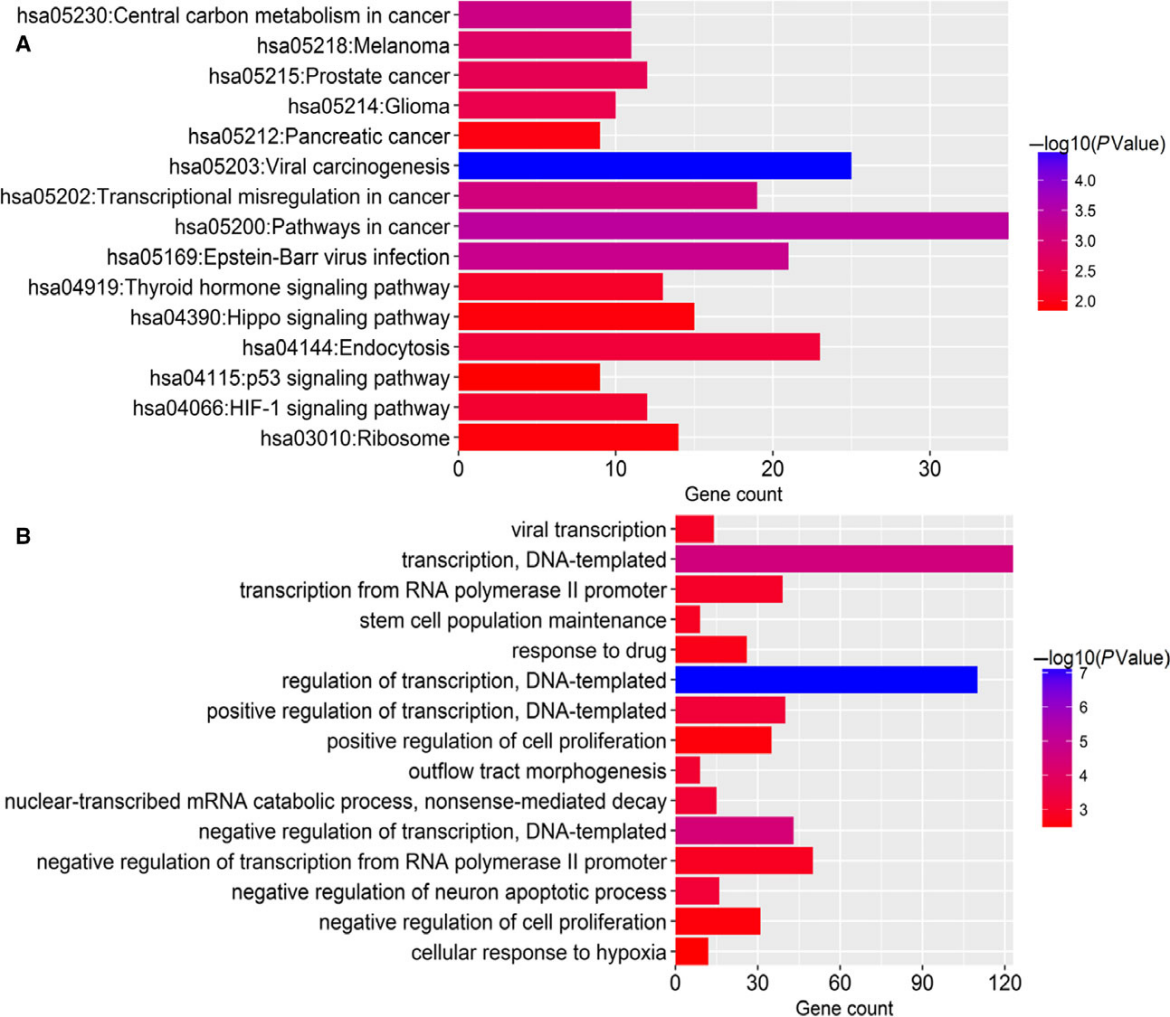
Functional enrichment analysis for predicted target genes of the six miRNA identified as independent predictors of HCC recurrence risk. (A) KEGG enrichment analysis. (B) GO enrichment analysis. The *x*‐axis represents the number of genes, and the *y*‐axis represents the GO terms and KEGG pathway names. The color indicates the *P*‐value.

As a preliminary investigation of the function of the six miRNA most accurate in predicting HCC recurrence, a total of 1113 genes targeted by these miRNA were first identified. A functional enrichment analysis of these genes showed significant enrichment of 92 GO processes and 25 KEGG pathways. The top 15 enriched KEGG pathways are shown in Fig. 6A. The results demonstrated that several cancer‐related pathways were highly activated in these patients with HCC, such as pathway in cancer, transcriptional misregulation in cancer, and the p53 signaling pathway. The enriched GO terms mainly included regulation of transcription, regulation of cell proliferation, and apoptosis, which have long been recognized as functions of miRNA. The top 15 enriched GO terms are shown in Fig. 6B. These results suggested that the six prognostic miRNA might be tightly associated with regulation of gene expression and critical cell biological functions.

## 4 Discussion

In this comprehensive analysis of miRNA sequencing data and its relation to recurrence in patients with HCC, we identified six miRNA that were closely associated with RFS in these patients. On the basis of the idea that a single miRNA is less sensitive and specific for prognostication than a combination of several miRNA (Xu *et al*., 2017), we developed a risk score formula based on these six miRNA. The six‐miRNA signature effectively divided patients in the training set into high‐ and low‐risk groups with significantly different RFS. We then successfully validated the signature in a testing set and in the entire set; the results indicated that the six‐miRNA signature had good reproducibility and reliability for prediction of RFS in patients with HCC. On further analysis, the six‐miRNA signature performed well in predicting recurrence at year 1, year 3, and year 5 and was an independent factor for predicting RFS in patients with HCC. The miRNA signature successfully classified patients with the same tumor stage into high‐ and low‐risk groups with significantly different RFS. A similar predictive ability was observed in patients with HCC stratified by age: Patients both in the younger dataset (age ≤ 65 years) and the older dataset (age > 65 years) were successfully divided into a high‐risk group and a low‐risk group with notably different prognoses. Similar results were observed when patients were stratified by tumor grade. Our six‐miRNA signature also was effective in stratifying patients with HCC into high‐ and low‐risk groups with significantly different OS. These results suggested that this miRNA signature might be helpful for clinical identification of patients who need more aggressive treatment, potentially prolonging their life.

It is well known that the initiation and progression of HCC is a long‐term process involving activation of key signaling pathways and dysregulation of cellular processes (Bupathi *et al*., 2015; Liu *et al*., 2014). Functional enrichment analysis of the six prognostic miRNA showed that most of the biological processes and pathways enriched are implicated in the development of HCC. Moreover, reports from other investigators indicate that four of the six miRNA play critical roles in human cancers. For example, miR‐139 has been reported to participate in a variety of biological processes, such as proliferation, apoptosis, invasion, and metastasis. Downregulation of miR‐139 was indicated to be associated with metastasis in HCC (Wong *et al*., 2011). A recent study showed that miR‐139‐5p could control cell growth, cell cycle, and apoptosis by targeting NOTCH1 in colorectal cancer (CRC) (Zhang *et al*., 2014). MiR‐139 could also function as a metastasis suppressor in CRC and may provide a therapeutic strategy for blocking CRC metastasis (Shen *et al*., 2012).

Several reports have revealed the mechanisms underlying the role of miR‐22 in development of cancers. MiR‐22 acts as a tumor suppressor through direct repression of MYCBP expression and subsequent reduction in oncogenic c‐Myc activities (Xiong *et al*., 2010). In gastric cancer, miR‐22 could inhibit tumor growth and metastasis by directly targeting MMP14 and Snail (Zuo *et al*., 2015). Interestingly, miR‐22 was reported to be downregulated in HCC, suppressing cell proliferation and tumorigenicity, and was correlated with more favorable prognosis of patients with HCC (Zhang *et al*., 2010), findings consistent with ours. A recent study indicated that vitamin D plays an anticancer role in CRC cells through suppressing proliferation, migration, and gene regulation by inducing the expression of miR‐22 (Alvarez‐Diaz *et al*., 2012), suggesting that this miRNA has a beneficial effect.

MiR‐210 has been widely investigated for the past few years and has most often been reported to be an oncomiR. In prostate cancer, miR‐210‐3p could promote cancer cell epithelial–mesenchymal transition and bone metastasis via the NF‐κB signaling pathway (Ren *et al*., 2017). Hypoxia‐induced miR‐210 enhances cancer cell viability by promoting proliferation and inhibiting apoptosis in epithelial ovarian cancer (Li *et al*., 2014). MiR‐210 was also identified as a biomarker or prognostic factor in breast cancer (Camps *et al*., 2008), clear cell renal cell carcinoma (Samaan *et al*., 2015), glioma (Lai *et al*., 2015), and head and neck cancer (Gee *et al*., 2010). MiR‐550a was implicated, by targeting RNF43, an inhibitor of Wnt/β‐catenin signaling, in promoting metastasis of CRC *in vitro* and *in vivo* (Wang *et al*., 2016). MiR‐550a also could act as a pro‐metastatic gene and directly targeted cytoplasmic polyadenylation element‐binding protein 4 in HCC (Tian *et al*., 2012).

Reports describing the function of miR‐3199‐2 and miR‐4732 in cancer are still rare. Our differential expression analysis indicates significant downregulation of these two miRNA in tumor tissues, suggesting their potential roles in tumorigenesis. Further investigation of the possible biological functions of these two miRNA at the cell level is warranted to expand our understanding of the molecular mechanism of HCC development. We have noticed that our findings are different from a recent study (Liu *et al*., 2017). The miRNA signature reported by Liu *et al*. is designed to predict the OS, but in our study, we focus on RFS. Moreover, the statistical analyses, *P*‐value setup, and inclusion criteria are different between the two studies. On the other hand, using Liu's methods, we have found a total of 24 miRNA (approximately 44% of all the miRNA identified in Liu's analysis) overlapped between the two studies.

These results, together with our own analysis, further strengthen the possibility that the six‐miRNA signature could be used effectively to predict disease course in HCC. Nevertheless, there may be some shortcomings to our study. First, there is a lack of experimental studies that might provide more convincing explanation of the biological implications and molecular mechanisms of these prognostic miRNA in liver cancer; second, a small proportion of results in the stratified survival analysis was not statistically significant but rather with trend difference, which may be attributed to the limited sample size after repeated grouping; third, independent cohorts from multicenter study in large population are required to validate the prognostic value of the miRNA signature before it can be applied to clinical practice.

## 5 Conclusion

In summary, after a comprehensive analysis, we have constructed a six‐miRNA signature that could serve as a reliable biomarker for stratifying risk of recurrence among patients with HCC. Further analysis revealed that the prognostic value of this miRNA signature was independent of other clinical features. Our study highlights the great potential of miRNA as tumor markers and therapeutic targets for patients with HCC.

## Conflict of interest

All authors have no conflict of interests.

## Author contributions

QHM, JXL, and FMB conceived and designed the study. FMB, HBZ, MNM, and CG accessed and analyzed the data. QHM, JXL, and FMB provided interpretation of the results. QHM, JXL, FMB, HBZ, and MNM wrote and revised the manuscript. All authors read and approved the final manuscript.

## Supporting information


**Fig. S1.** Survival analysis of HCC patients in the training set stratified by stage, age and tumor grade.
**Fig. S2.** Survival analysis of HCC patients in the testing set stratified by stage, age and tumor grade.
**Fig. S3.** Association between six‐miRNA signature and OS of HCC patients in different sets.
**Fig. S4.** Stratified analysis of association between six‐miRNA signature and OS of all HCC patients.
**Fig. S5.** Stratified analysis of association between six‐miRNA signature and OS of HCC patients in the training set.
**Fig. S6.** Stratified analysis of association between six‐miRNA signature and OS of HCC patients in the testing set.
**Fig. S7.** Performance assessment of the six‐miRNA signature by survival ROC analysis.
**Table S1.** Baseline demographic and clinical features of HCC patients in training and testing set.
**Table S2.** Multivariable Cox regression analysis of OS in HCC patients in the training, testing, and entire sets.
