# Supplementary material for: A novel RNA sequencing‐based miRNA signature predicts with recurrence and outcome of hepatocellular carcinoma
Source: Mol Oncol. 2018 May 21;12(7):1125–37. doi: 10.1002/1878-0261.12315 (PMC6026871; doi:10.1002/1878-0261.12315)
Supplement: Supplementary file 1 — Fig. S1. Survival analysis of HCC patients in the training set stratified by stage, age and tumor grade. Fig. S2. Survival analysis of HCC patients in the testing set stratified by stage, age and tumor grade. Fig. S3. Association between six‐miRNA signature and OS of HCC patients in different sets. Fig. S4. Stratified analysis of association between six‐miRNA signature and OS of all HCC patients. Fig. S5. Stratified analysis of association between six‐miRNA signature and OS of HCC patients in the training set. Fig. S6. Stratified analysis of association between six‐miRNA signature and OS of HCC patients in the testing set. Fig. S7. Performance assessment of the six‐miRNA signature by survival ROC analysis. Table S1. Baseline demographic and clinical features of HCC patients in training and testing set. Table S2. Multivariable Cox regression analysis of OS in HCC patients in the training, testing, and entire sets. [file MOL2-12-1125-s001.pdf]

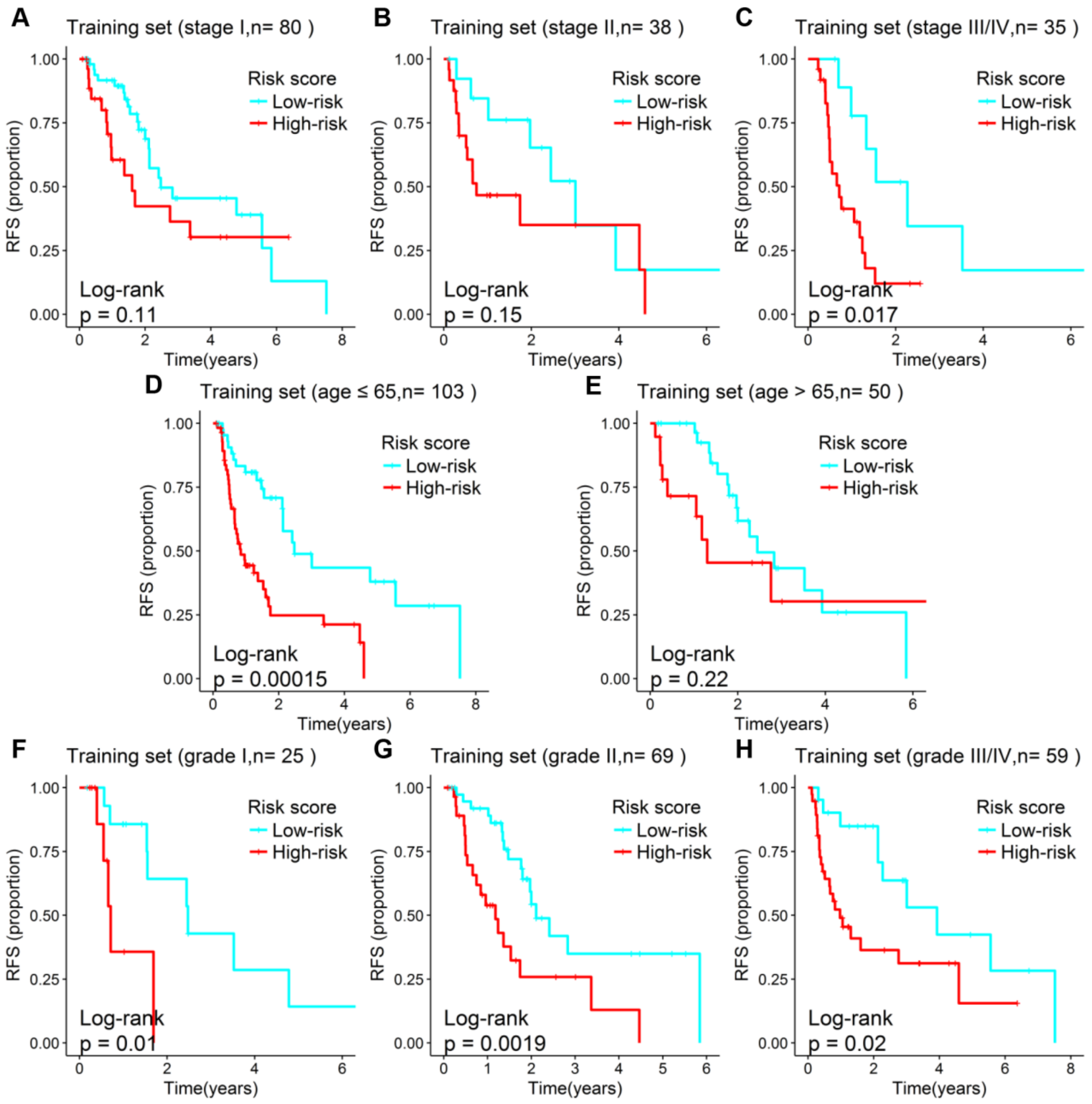

Fig. S1: Survival analysis of HCC patients in the training set stratified by stage, age and tumor grade. Survival analysis compared recurrence-free survival (RFS) by recurrence risk (high vs. low) stratified by clinical characteristics**.** Kaplan-Meier curves for patients with stage I HCC (n=80) (**A**), patients with stage II HCC (n=38) (**B**), patients with stage III/IV HCC (n=35) (**C**), patients aged 65 years or younger (n=103) (**D**), patients aged older than 65 years (n=50) (**E**), patients with grade I tumor (n=25) (**F**), patients with grade II tumor (n=69) (**G**), patients with grade III/IV tumor (n=59) (**H**). The “+” symbols in the panel indicate censored data.

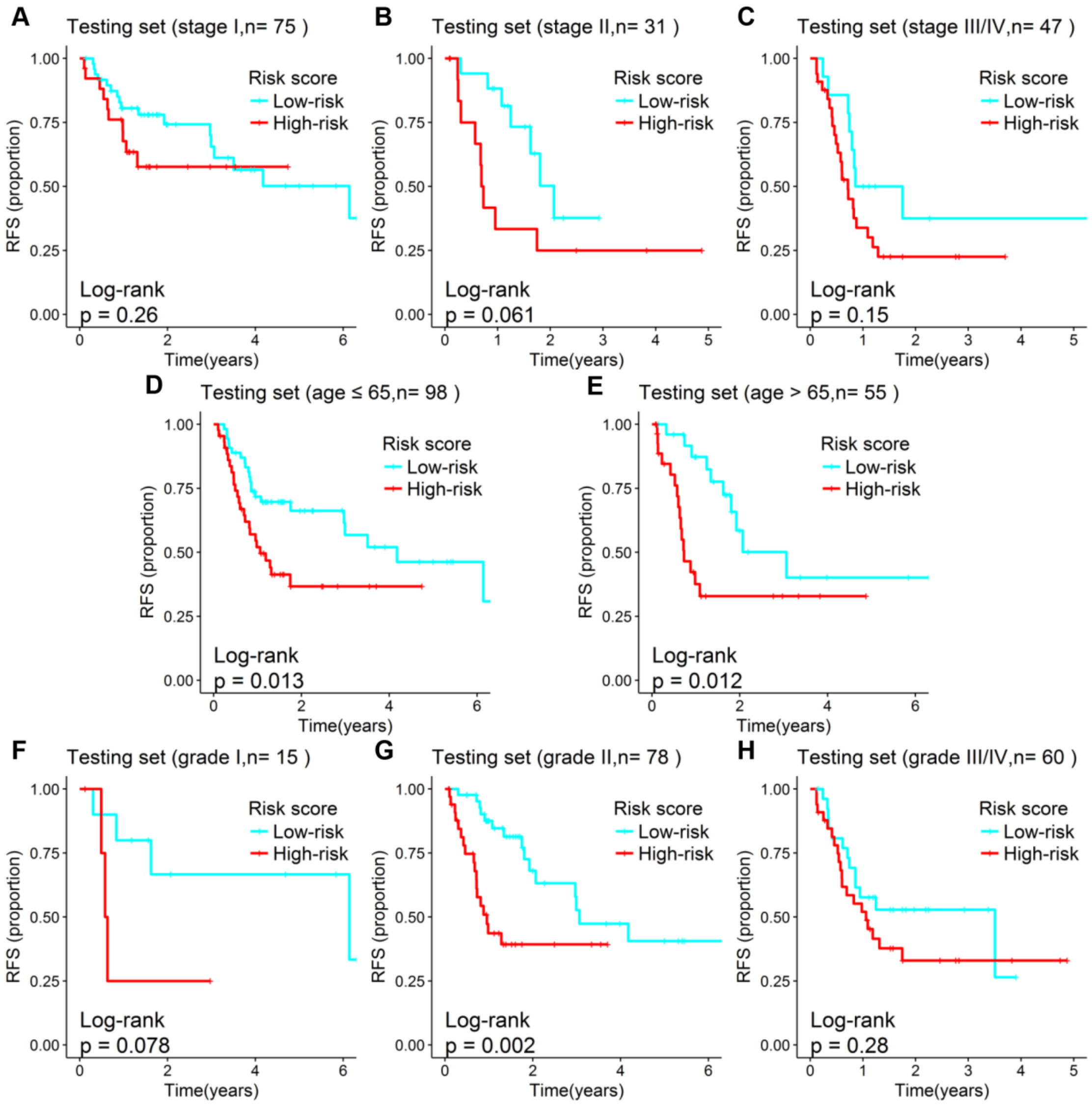

Fig. S2: Survival analysis of HCC patients in the testing set stratified by stage, age and tumor grade. Survival analysis compared recurrence-free survival (RFS) by recurrence risk (high vs. low) stratified by clinical characteristics. Kaplan-Meier curves for patients with stage I HCC (n=75) (**A**), patients with stage II HCC (n=31) (**B**), patients with stage III/IV HCC (n=47) (**C**), patients aged 65 years or younger (n=98) (**D**), patients aged older than 65 years (n=55) (**E**), patients with grade I tumor (n=15) (**F**), patients with grade II tumor (n=78) (**G**), patients with grade III/IV tumor (n=60) (**H**). The “+” symbols in the panel indicate censored data.

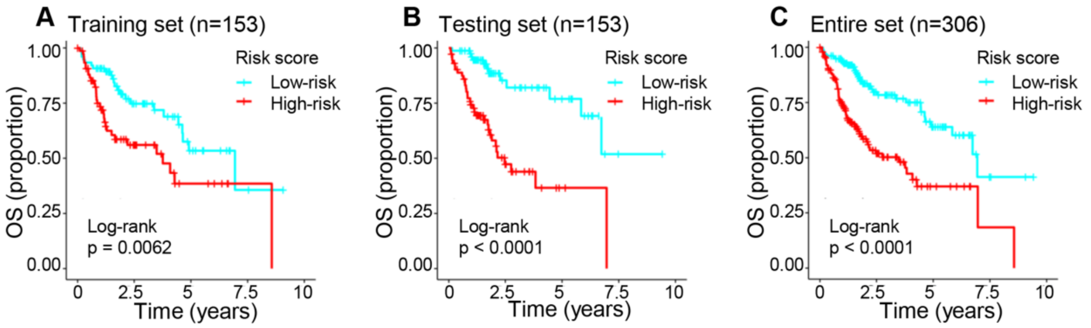

Fig. S3: Association between six-miRNA signature and OS of HCC patients in different sets. Kaplan-Meier curves of OS between high- and low-risk patients in training set (**A**), testing set (**B**), and entire TCGA set (**C**). The "+" symbols in the panel indicate censored data.

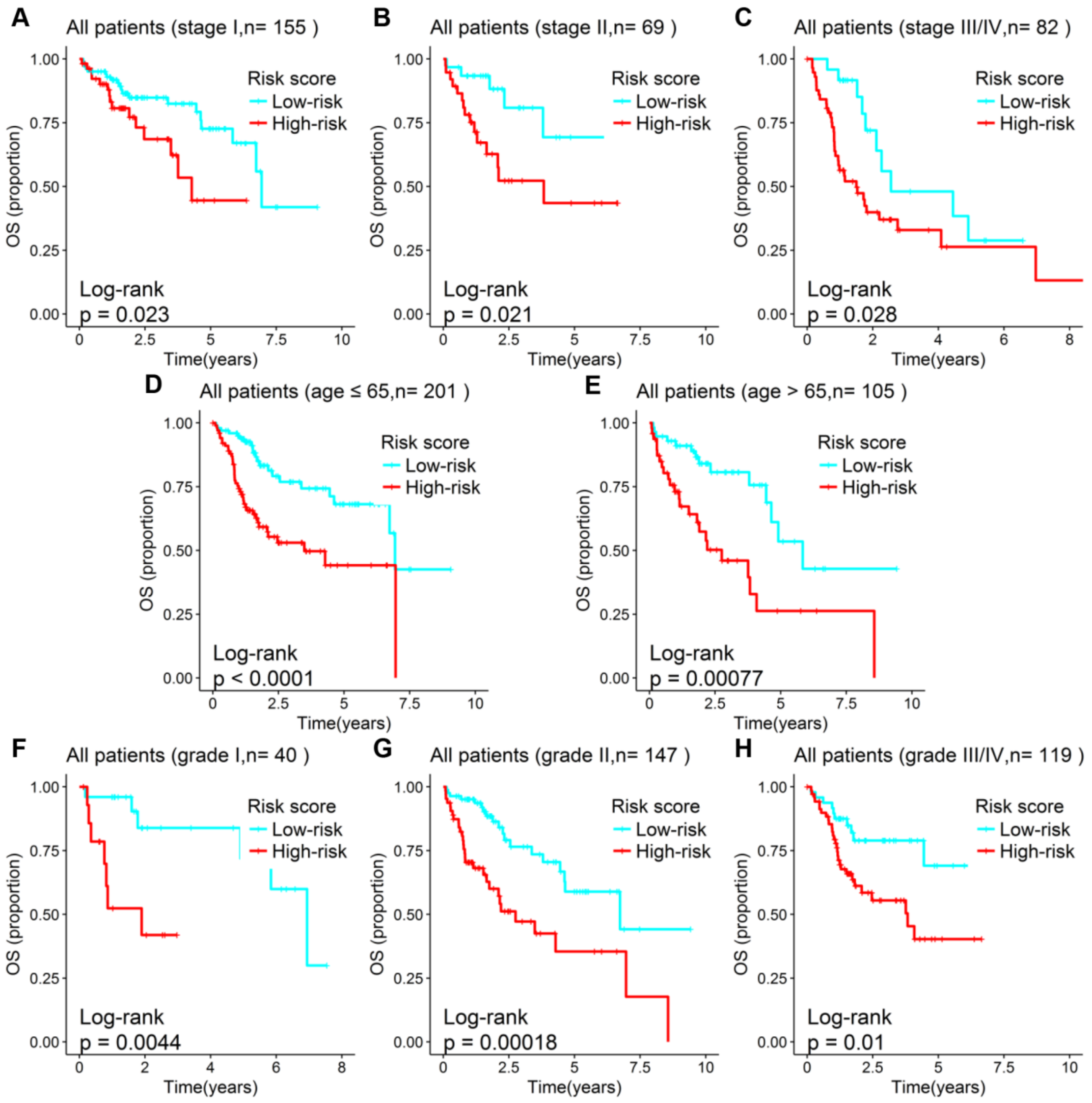

Fig. S4: Stratified analysis of association between six-miRNA signature and OS of all HCC patients. Kaplan-Meier curves for patients with stage I HCC (n=155) (**A**), patients with stage II HCC (n=69) (**B**), patients with stage III/IV HCC (n=82) (**C**), patients aged 65 years or younger (n=201) (**D**), patients aged older than 65 years (n=105) (**E**), patients with grade I tumor (n=40) (**F**), patients with grade II tumor (n=147) (**G**), patients with grade III/IV tumor (n=119) (**H**). The "+" symbols in the panel indicate censored data.

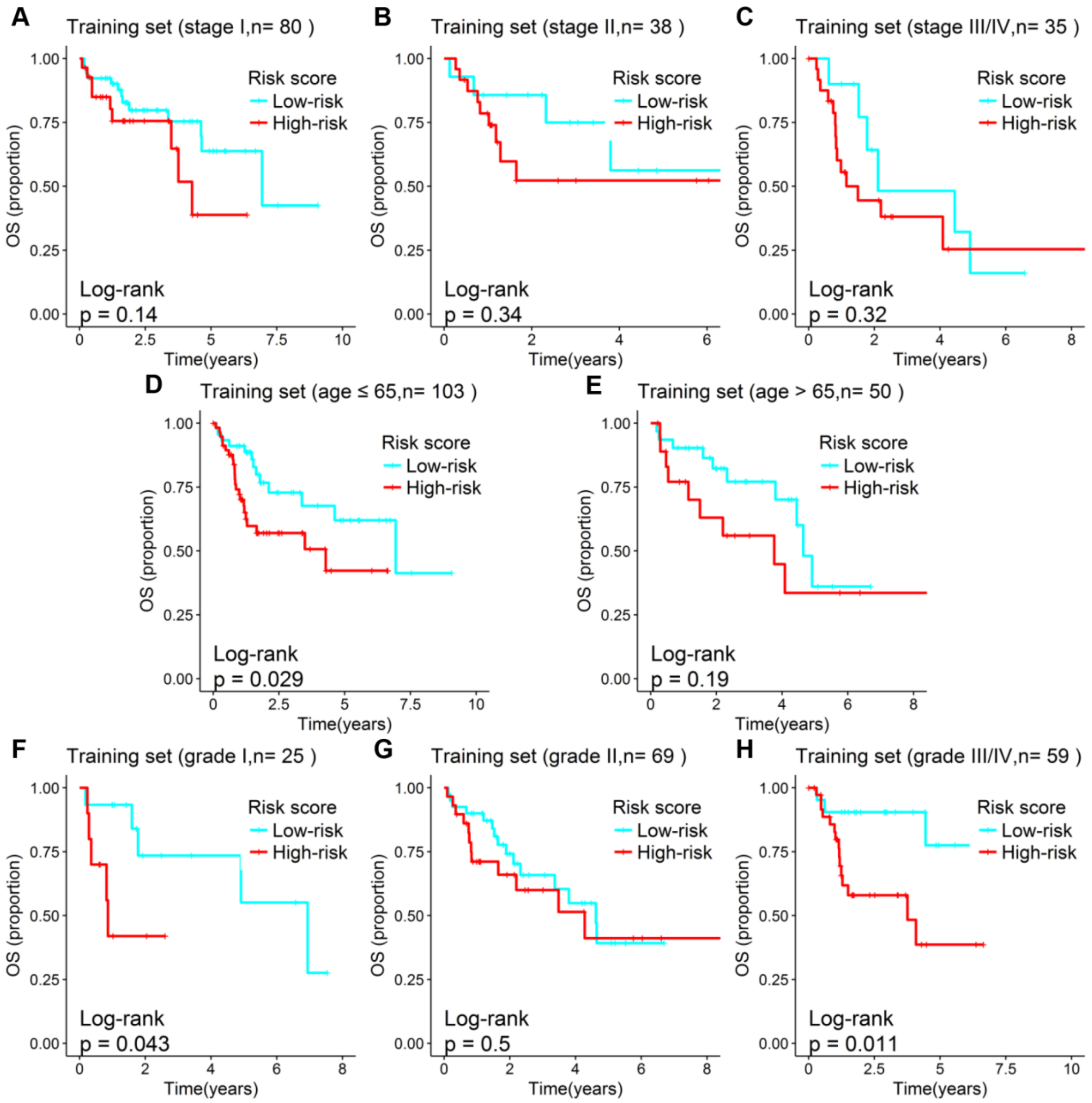

Fig. S5: Stratified analysis of association between six-miRNA signature and OS of HCC patients in the training set. Kaplan-Meier curves for patients with stage I HCC (n=80) (**A**), patients with stage II HCC (n=38) (**B**), patients with stage III/IV HCC (n=35) (**C**), patients aged 65 years or younger (n=103) (**D**), patients aged older than 65 years (n=50) (**E**), patients with grade I tumor (n=25) (**F**), patients with grade II tumor (n=69) (**G**), patients with grade III/IV tumor (n=59) (**H**). The "+" symbols in the panel indicate censored data.

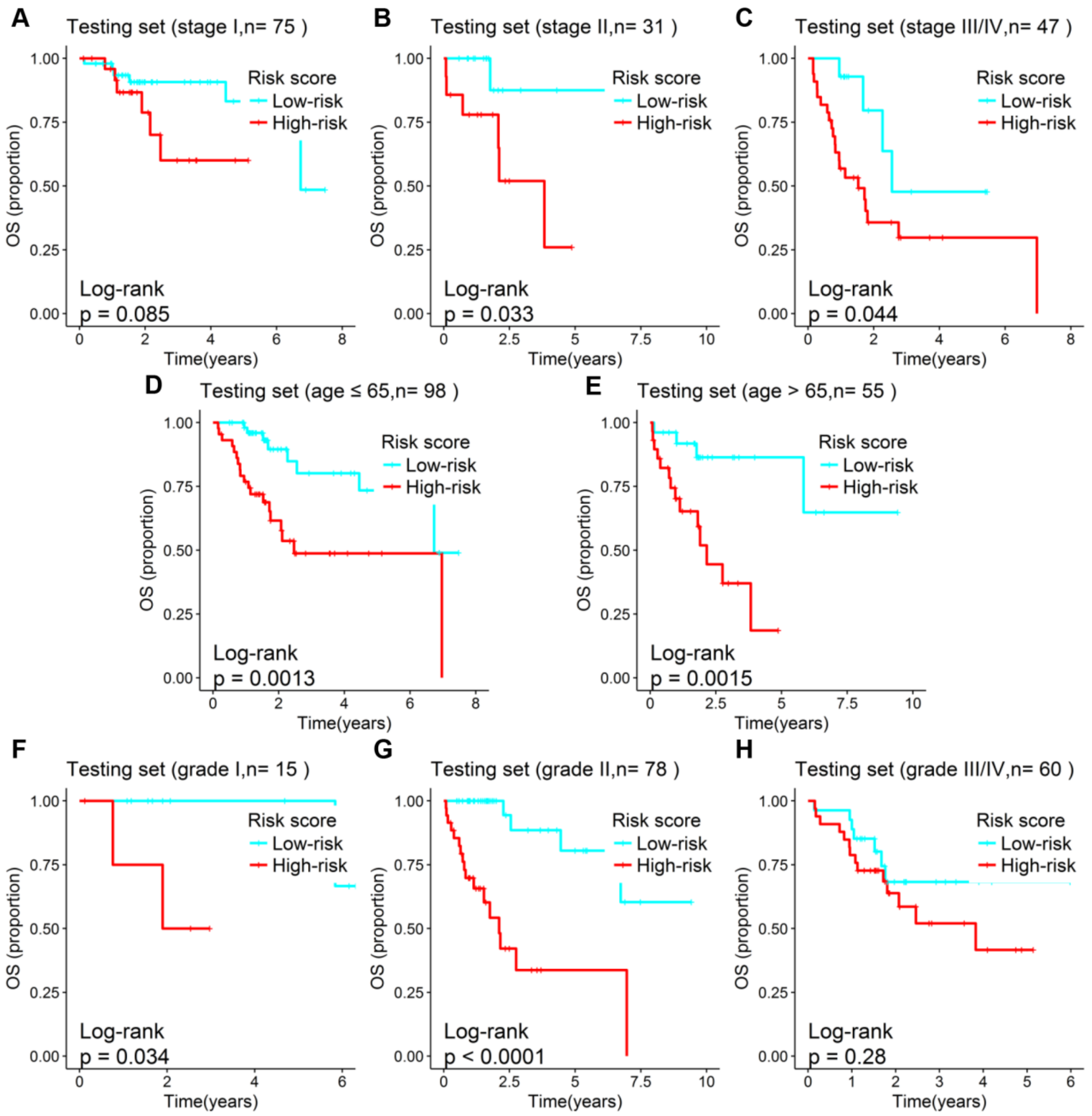

Fig. S6: Stratified analysis of association between six-miRNA signature and OS of HCC patients in the testing set. Kaplan-Meier curves for patients with stage I HCC (n=75) (**A**), patients with stage II HCC (n=31) (**B**), patients with stage III/IV HCC (n=47) (**C**), patients aged 65 years or younger (n=98) (**D**), patients aged older than 65 years (n=55) (**E**), patients with grade I tumor (n=15) (**F**), patients with grade II tumor (n=78) (**G**), patients with grade III/IV tumor (n=60) (**H**). The “+” symbols in the panel indicate censored data.

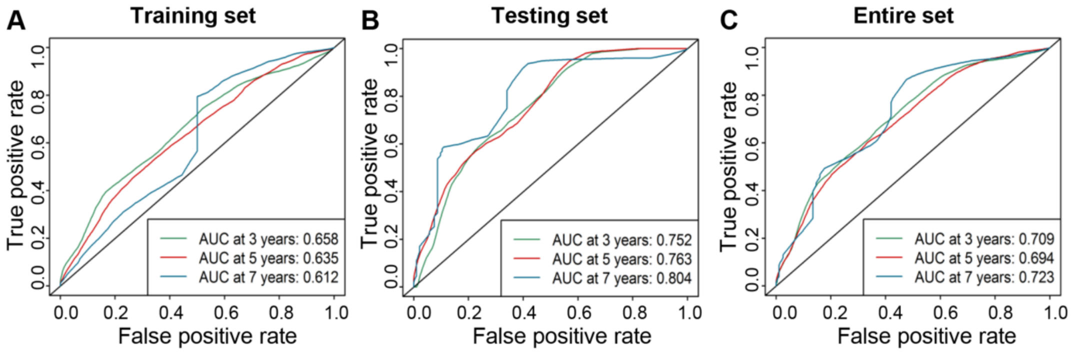

Fig. S7: Performance assessment of the six-miRNA signature by survival ROC analysis. Receiver operating characteristic (ROC) analysis of the six-miRNA signature for prediction of overall survival (OS) at 3, 5, and 7 years in the training set (**A**), the testing set (**B**), and the entire set (**C**).

Table S1. Baseline demographic and clinical features of HCC patients in training and testing set

Characteristic		Training set (n=153)	Testing set (n=153)	p-value
**Sex**				0.901[a]
	Male	108	106	
	Female	45	47	
**Age, years (mean±SD)**		58.8 ±13.0	58.6±12.8	0.884[b]
**Tumor stage**				0.42[a]
	Stage I	80	75	
	Stage II	38	31	
	Stage III	33	45	
	Stage IV	2	2	
**Tumor grade**				0.321[a]
	G I	25	15	
	G II	69	78	
	G III	52	55	
	G IV	7	5	
**AJCC TNM staging system**				
**T stage**				0.458[a]
	T1	80	76	
	T2	39	32	
	T3	29	40	
	T4	5	5	
**N stage**				0.624[a]
	N0	113	118	
	N1	1	2	
	Nx	39	33	
**M stage**				0.808[a]
	M0	120	114	
	M1	2	2	
	Mx	31	37	

[a]chi-square test; [b]Student *t*-test; SD: standard deviation

HCC, hepatocellular carcinoma; AJCC, American Joint Committee on Cancer

Tumor stage: AJCC pathological stage; Tumor grade: neoplasm histologic grade

T stage: tumor size; N stage: lymph node involvement; M stage: Metastasis status

Table S2. Multivariable Cox regression analysis of OS in HCC patients in the training, testing, and entire sets

**Characteristic**		**Multivariable analysis**		
		**HR**	**95% CI of HR**	**p-value**
**Training set (n=153)**				
**Risk score**		1.594	1.272-1.996	**<0.0001**
**Sex**	Female/Male	1.073	0.605-1.904	0.810
**Age, years**	≥65 /<65	1.028	0.572-1.848	0.925
**Tumor stage**	III.IV/I.II	1.860	1.007-3.436	0.048
**Tumor grade**	III.IV/I.II	0.96	0.384-1.259	0.231
**T stage**	T3.T4/T1.T2	NA	NA	NA
**N stage**	non-N0/N0	0.921	0.421-2.017	0.837
**M stage**	non-M0/M0	1.706	0.791-3.678	0.173
**Testing set (n=153)**				
**Risk score**		1.622	1.227~2.143	**0.0006**
**Sex**	Female/Male	0.950	0.496-1.819	0.878
**Age, years**	≥65 /<65	1.364	0.711-2.616	0.350
**Tumor stage**	III.IV/I.II	0.641	0.074-5.551	0.687
**Tumor grade**	III.IV/I.II	1.122	0.569-2.210	0.740
**T stage**	T3.T4/T1.T2	5.508	0.604-50.258	0.130
**N stage**	non-N0/N0	1.442	0.649-3.203	0.369
**M stage**	non-M0/M0	2.062	0.973-4.370	0.059
**Entire set (n=306)**				
**Risk score**		1.646	1.393~1.945	**<0.0001**
**Sex**	Female/Male	0.998	0.658-1.512	0.991
**Age, years**	≥65 /<65	1.110	0.734-1.677	0.622
**Tumor stage**	III.IV/I.II	0.599	0.076-7.749	0.627
**Tumor grade**	III.IV/I.II	0.860	0.556-1.331	0.500
**T stage**	T3.T4/T1.T2	4.011	0.500-32.137	0.191
**N stage**	non-N0/N0	1.206	0.703-2.701	0.496
**M stage**	non-M0/M0	1.740	1.033-2.932	0.037

HR, hazard ratio; CI, confidence interval

HCC, hepatocellular carcinoma; OS, overall survival

Tumor grade: neoplasm histologic grade; Tumor stage: AJCC pathological stage

T stage: tumor size; N stage: lymph node involvement; M stage: Metastasis status
